# The changing roles of scientific journals

**DOI:** 10.1128/mbio.02515-24

**Published:** 2024-10-04

**Authors:** Arturo Casadevall, Lorraine F. Clark, Ferric C. Fang

**Affiliations:** 1 Department of Molecular Microbiology and Immunology, Johns Hopkins School of Public Health, Baltimore, Maryland, USA; 2 American Society for Microbiology, Washington, DC, USA; 3 Departments of Laboratory Medicine, Pathology and Microbiology, University of Washington, Seattle, Washington, USA; Albert Einstein College of Medicine, Bronx, New York, USA

**Keywords:** publishing, scientific, journals

## Abstract

After centuries of relative stability, the scientific publishing world has undergone tremendous disruption and change during the first decades of the 21st century. The causes for disruption can be traced to the information revolution, which brought such benefits as rapid publication, greater connectivity, and ready access to large databases, along with less desirable practices including image manipulation, plagiarism, and other ethical transgressions. The information revolution has driven the proliferation of journals, expansion of for-profit academic publishing, and empowerment of the open-access movement, each of which has exerted new financial pressures on traditional publishing models. As journals became the focal point for ethical concerns in science, they have adapted by increasing the scope of their duties, which now include archiving of data, enforcement of good practices, establishment of standards for rigor, and training the next generation of reviewers and editors. Here, we consider the seismic changes occurring in scientific publishing and place them into the context of a rapidly changing landscape of scientific and publishing norms.

## INTRODUCTION

Older scientists can still recall that submitting a scientific paper to a journal involved typing a cover letter, making three photocopies of the paper, and placing it in a large envelope for expedited mail delivery. Today, submitting a paper to a journal is a complex and tedious online process that can take from hours to days as authors are required to meet many journal requirements, deposit data sets in public databases, and sometimes even include primary data for review and storage at the journal.

What happened? On one hand, the creation of the internet made electronic submissions possible, and the low cost of computer memory storage facilitated the transfer of high-resolution images and data from the laboratory to the journal. The digital revolution has expedited review and publication processes, reduced paper consumption, and facilitated global access to information (1). On the other hand, these advances have created new problems that have forced journals to take a proactive role in ensuring the reliability and validity of submissions. Inappropriate image manipulation, the rise of predatory journals, the open-access movement, and the need for digital archives have increased pressures on society-sponsored journals. Hence, in less than a generation, the process of scientific publishing and the role of journals have changed dramatically. Here, we review some of these changes and the causes that drove them.

In 1665, the *Journal des Sçavans* and *Philosophical Transactions of the Royal Society* were launched in France and England, respectively (2), At that time, Henry Oldenburg and Robert Hooke described journals as fulfilling the following functions: registration, certification, dissemination, and archiving (3). The roles of scientific journals did not change much over the next three and a half centuries, being largely limited to receiving manuscripts, vetting them through some sort of peer review, and publishing them to disseminate scientific information. Although journals have differed in scope, practices, and reputation, their primary role over more than three centuries has been to serve as gatekeepers for information, publishing only scientific information that met standards of rigor, respectability, and peer acceptability.

In the early decades of the 21st century, the roles of journals changed as they acquired new responsibilities (4, 5). The new responsibilities were driven largely by reproducibility problems with publications; the rise of the open-access/open science movements; increased awareness of the importance of diversity, equity, and inclusion (DEI); and technological advances to which journals and the entire scientific enterprise have been forced to adapt. In addition, the economics of scientific publishing have changed in dramatic ways. Scientific publishing has become an oligopoly, with five publishers (Elsevier, Wiley, Springer, Taylor & Francis, and Sage) now accounting for more than half of scientific publishing worldwide (6). The open-access movement has further complicated the economics of publishing, with a collateral effect of marginalizing the role of librarians and throwing open the gates to predatory publishers, who exploit the open-access model (7). By “predatory,” we mean the publishers of journals that appear to be legitimate but misrepresent their practices, including falsely claiming to provide peer review, misrepresenting publication costs, and inventing editorial boards to make a profit from unsuspecting authors (8). A consensus definition has defined predatory journals and publishers as “entities that prioritize self-interest at the expense of scholarship and are characterized by false or misleading information, deviation from best editorial and publication practices, a lack of transparency, and/or the use of aggressive and indiscriminate solicitation practices” (9).

The primary function of many commercial journals is financial gain for the publisher while publishing scientific literature. In contrast, scientific societies are nonprofit organizations that continue to publish journals with the primary goal of facilitating the communication of important scientific results in their field. This creates conflicting incentives: while societies are motivated to keep costs for authors and readers as low as possible, commercial publishers are motivated to keep article processing charges (APCs) and subscription fees as high as possible (6). Funds generated by society journals are used not only to support the operation of the journal but also to advance other missions of the society that advance the field (10). In contrast, funds generated by commercial journals, whether from article processing charges or subscriptions, end up entirely in private hands, leaving the scientific enterprise (10–12). Costs have skyrocketed and created a >$25 billion (USD) industry. With steeply rising subscription and article processing charges, large publishers have been making record profits, and a large percentage of public expenditures on research are now going to these companies (13). The conflict in goals and incentives can be seen in recent clashes over pricing between the Dutch publisher Elsevier and academic institutions and governments, which led to some scientists temporarily losing access to thousands of journals (14, 15). The high market concentration of scientific publishing and anti-competitive practices have prompted calls for intervention and reform (16). Meanwhile, scientific societies and commercial publishers continue to compete on an uneven battlefield. Against this background, we consider some of the many ways that the roles of journals have evolved in the past two decades.

### Storage of scientific information

For centuries, journals preserved the scholarly record by creating “versions of record” (final published versions of an article). Printed copies, copyright laws, and policies preserved this system (17). Now, these roles are threatened, as repositories have become virtual. Faced with image problems and questions about the reliability of scientific results, journals have become increasingly proactive in ensuring that primary data are available. This has taken the form of requiring photographs of gels, databases, etc. In 2014, *PLoS* (the Public Library of Science) announced a policy requiring authors to make the data supporting their research to be publicly available (18), and many journals, including those published by the American Society for Microbiology (ASM), now have similar data policies (19). Hence, data storage, a task that previously was the responsibility of the laboratories that generated the data, has increasingly been assumed by journals. One technological advance that has facilitated this transition is the increasingly low cost of digital data storage. Nevertheless, the costs of preserving data in perpetuity are considerable and represent a potential vulnerability as the scientific record has moved from libraries onto decentralized servers.

### Arbiters of quality

Whereas the arbiters of information quality throughout much of scientific history have been authors, reviewers, and readers, many journals have increasingly taken actions to ensure the quality of their published work. Early examples of journals developing standards to improve the quality of published information were the efforts by clinical journals to standardize the reporting of clinical study data and statistical methodology (20). In 2013, the journal *Nature* introduced a checklist “intended to prompt authors to disclose technical and statistical information in their submissions, and to encourage referees to consider aspects important for research reproducibility” (21). Beginning in 2012, *The Journal of Clinical Investigatio*n instituted a policy requiring submission of uncropped images of Western blots along with the finished manuscript (22). In an internal audit carried out over 7 months in 2018–2019, that journal screened 200 manuscripts and found that 28.5% had statistical problems, 21% had issues with blots, and 27.5% contained problematic images, which led to corrections prior to publication and rescinding of two acceptances (22). Many journals are also utilizing plagiarism detection software and image analysis tools to examine submitted papers prior to publication. Ensuring integrity of publications has become a critical issue for publishers, as illustrated by the development of the Integrity Hub hosted by the not-for-profit organization STM, which provides publishers with a platform to check for research integrity concerns in submitted articles and to collaborate with each other to create manuscript inspection applications (23).

### Improvement of scientific standards

In some cases, journals do not merely act as arbiters of quality, but by defining standards for publication, they raise the bar for scientific practice. For example, as the inclusion of digital image data became increasingly common in the early 2000s, the *Journal of Cell Biology* took the lead in proposing guidelines for image preparation and presentation that continue to be widely cited today (24). Another way in which journals have taken an active role to enhance science quality is by improving the use and interpretation of statistical methods in data analysis (25). By providing guidance to authors and requiring statistical rigor as a condition for publication, journals are raising the standards of science.

### Promoting new research findings

With more than five million new scholarly articles now being published each year, it is largely the journals that dictate which articles receive attention. This is a two-edged sword, as the peer review process is imperfect, and the most highly selective journals all too often seem to favor impact over importance and rigor (26–28). Nevertheless, scientists are increasingly reliant on journals to filter the literature and prioritize what they should read (29). Accordingly, journals are increasingly focused on practices to ensure and enhance discoverability of their content (e.g., metadata, syndication, use tracking, and analytics).

### Assessment of researcher productivity

Journals are widely used as a source of metrics to assess the productivity of individual scientists, which has an enormous impact on the ability of scientists to gain professional recognition, promotion, and funding. Although bibliometrics are easy to measure and provide the illusion of precision and objectivity, their shortcomings as indicators of scientific output quality are well known (30, 31). The obsession with bibliometrics in turn has had an adverse impact on scientific misconduct and added to the burden on journals as guardians of research integrity. The use of citations to measure researcher productivity encourages an emphasis on publication venue rather than on the work itself and values impact over importance (26). Concerns about the mismeasure of science and scientists lead to the 2013 Declaration on Research Assessment (DORA) and the 2015 Leiden Manifesto, which encourage a broader range of research metrics and the importance of assessing research on its own merits, rather than on the reputation of the journal in which it is published (32, 33). Excessive emphasis on citation metrics has fueled detrimental trends in science such as “salami slicing,” inflated self-citations, citation cartels, gift authorships, and inflated multi-authorship (34). Vincent LaRiviere and colleagues have also pointed out that the “counting of papers. . . reinforces the control of commercial publishers on the scientific community (6).”

### Ethics and misconduct arbitration

When questions about a scientific paper arise, journals are often the first to receive complaints. Journals may refer concerns about the ethics, quality, and reliability of published results to authors and their home institutions for adjudication. However, this process is lengthy and consumes a tremendous amount of time and resources. When significant concerns are identified in published articles, journals must make decisions on whether to seek a correction, issue a notice of concern, or retract the paper. In recent years, the biomedical sciences found itself facing a reproducibility crisis (35), a retraction epidemic driven largely by scientific misconduct (36), including the rise of paper mills and problematic images in as many as 4%–6% of published papers (37, 38). Although these problems originated primarily in the research laboratories that submitted manuscripts for publication, journals found themselves at the epicenter of the crisis since many scientists and lay people, fairly or not, blamed the peer review and publishing system for publishing flawed work. Furthermore, questions about published manuscripts directed to the journals began to consume their resources. For example, an audit of the experience of the journal *Molecular and Cell Biology* revealed that adjudicating and resolving inappropriate image duplications required 6 hours on average for each problematic figure after publication, whereas problem identification and correction before publication took only 30 minutes (38). In the early 2020s, manuscripts published by so-called “paper mills,” organizations that sell authorship to papers, often containing fabricated data, led to hundreds of retractions at various journals (39). For example, in 2021, The Royal Society of Chemistry retracted 69 articles they believed were due to paper mill activity (40). Consequently, journal practices have been instituted to prevent problems with published articles, which require greater scrutiny of submitted and accepted manuscripts prior to publication. The Committee on Publications Ethics (COPE) serves as a resource to publishers, editors, and institutions to address publication concerns and offer best practice guides, educational courses, and an opportunity to submit publication ethics issues to a forum for further discussion (41). Many publishers, including ASM, now have a team of publication ethics experts who review and consult on publication integrity issues with journal staff, editors, and authors (42).

### Training peer reviewers

Journals rely on scientists for peer review and advice about the suitability of a particular submission for publication. However, the quality of peer review can be uneven, and major journals are presently experiencing a reviewer crisis because of the increasing difficulty finding experts willing to volunteer their time to review a paper. In recent years, journals and scientific organizations have taken an interest in improving peer review, recognizing a gap in the training of scientists in this important activity (see, for example, reference 43). The Genetics Society of America has developed online resources for peer review (44), and online courses teach basic elements of peer review (45). The ASM has gone further in creating an Early-Career Editorial Board (ECB) at the journal *mBio*, in which scientists at the postdoctoral, assistant professor, or equivalent stages of their careers are given the opportunity to participate in peer review with supervision by experienced journal editors. To complement this experience, members of the ECB attend training sessions hosted by journal staff that cover various aspects of peer review (46).

### Training editors

In addition to training reviewers, the need to educate editors beyond knowing how to work with a particular submission system is being recognized. Editors are essential to journals, as they provide expertise in their respective fields and experience in the publishing process. For example, the editor in chief of *Applied and Environmental Microbiology*, Gemma Reguera, instituted monthly editors’ meetings to raise awareness about unconscious biases that can affect the peer review process (47). At the journal *mSystems,* editors regularly discuss manuscript content and manuscript decisions to calibrate their assessments of manuscript scope and quality. Editor training is also available in the form of webinars or training courses, such as those offered by Springer Nature (48) and Elsevier (49). Educated editors are essential for successful journals, and such programs provide guidance for day-to-day journal management as well as helping formulate journal strategy and foster community engagement.

### Research on the workings of science

Publication is the final step in the process of scientific research. In the process of handling submissions, arranging peer review, and publishing articles, journals generate a tremendous amount of information about the workings of science. Unfortunately, this information is seldom made publicly available, where it might be analyzed to improve the understanding about the workings of science. However, a new trend is emerging in which journals are analyzing and publishing their experiences and are emerging as sources of primary information on a variety of scientific processes. The journals *mBio* (37), *Molecular and Cellular Biology* (38), and the *Journal of Clinical investigation* (22, 50), among others, have published papers reporting their experiences with inappropriate image duplications. Ada Hagan and colleagues analyzed submission and outcome patterns at ASM journals and found that women were underrepresented as authors and more likely to receive a negative editorial decision (51). Another study examined papers in ASM journals to understand the reasons why authors sometimes share first authorship (52).

### Roles of journals that distinguish them from preprint servers

With the rise of preprints, some have questioned whether journals have become obsolete as a means of disseminating scientific information. The use of preprints skyrocketed during the COVID-19 pandemic, as there was a need for the rapid communication of medical and scientific information to solve the public health crisis. Although some questioned the balance between benefits and risks involved in the rapid reporting of information that has not been peer-reviewed (53), a retrospective comparison of clinical trial results first presented as preprints and then published in peer-reviewed journals found no compelling evidence that results in preprints were less reliable (54). Hence, preprint servers have evolved to facilitate the rapid dissemination of scientific information, while journals continue to maintain their role in quality control through peer review. However, the new roles for journals described herein point to an increasing set of tasks and responsibilities relating to the publication of peer-reviewed papers that further differentiate their functions from those of preprint servers.

### Promotion of diversity and inclusion

The scholarly publishing community has made diversity and inclusion important priorities in recent years, spurred in part by the killing of George Floyd in early 2020 and ensuing discussions around racism (55, 56). In the same year, the Joint Commitment for Action on Inclusion and Diversity in Publishing was developed, a global cross-organizational initiative spearheaded by the Royal Society of Chemistry (RSC). The goal of this initiative is for members to work together to enact positive change within scholarly publishing. As of 2024, 56 publishing organizations are collaborating on the Joint Commitment, representing over 15,000 journals. Members of the initiative have agreed to a set of four commitments: understanding their research communities via self-reported data; ensuring inclusive representation of authors, reviewers, and editors; sharing resources to improve representation and inclusion; and creating a set of minimum standards for scholarly publishing (57). ASM journals have been making efforts to ensure diversity and inclusion as part of their editorial goals and processes (58). This includes utilizing open calls to identify new members for editorial boards to achieve gender parity and greater geographic diversity, as well as publishing dedicated collections to highlight contributions from certain groups (see, for example, the ASM Journals collection focused on diversity, equity, and inclusion [59]). The ASM journal *Applied and Environmental Microbiology* has adopted specific initiatives to ensure best practices in diversity, equity, and inclusion, including a merit-based process for adding new reviewers to the Editorial Board and renewing appointments of existing board members (47).

### Diversification of peer review models

Modern peer review arose in the late 20th century, and journals most commonly use a single anonymized peer review system in which reviewer identities are concealed from authors (60). Critics of this model are concerned by the biases that it introduces, including the “Matilda effect” in which manuscripts by male first authors are considered of higher quality. (The “Matthew effect” describes the phenomenon in which established researchers benefit from their greater name recognition, and biases result from the reputation of authors’ affiliations [61].) Many journals now offer alternative peer review models, such as double-anonymized peer review (DAPR), in which both author and reviewer identities are masked; triple anonymized, in which all parties’ identities including those of editors are concealed; and open peer review, in which all parties’ identities are known to each other (62). Such approaches can improve peer review by minimizing conscious and unconscious biases. For example, double-blind anonymous peer review can reduce gender, geographic, and linguistic biases (63). At ASM journals, single anonymized peer review is still the most widely used system, although some ASM journals offer additional options (64). For example, *mSystems* and *Microbiology Spectrum* offer open peer review, with reviewer identities made visible if the reviewer agrees. In addition, the entire content of a reviewer’s report may be published if the author agrees, and reviewer names are published if the reviewers agree. Recently, the *Journal of Bacteriology* announced that it would offer double-anonymized peer review (65). The development and adoption of new peer review models beyond the traditional single-anonymized route illustrates ongoing efforts by journals to increase the fairness of the peer review process.

### Diversification of publication models

In the past, access to scientific articles was controlled by journal subscriptions, which are generally managed by librarians. In 1991, Paul Ginsparg at Los Alamos National Laboratory established the arXiv repository, which made physics preprints freely available, an event that is considered to mark the beginning of the open-access movement (66). The open-access (OA) model requires authors to pay a publication fee (article processing charge [APC]) in exchange for the article being made free to access upon publication (67). BioMedCentral was the first commercial publisher to launch a series of open-access journals in the year 2000 in biology and medicine (68). Open access has become increasingly popular since then, and the Directory of Open Access Journals (DOAJ) featured 20,734 indexed journals as of August 2024 (69), with about half of all newly published papers now published via this route (70). The increase in open-access publications has been driven largely by recent funders’ stipulations that require research supported by their grants to be freely available upon publication. An example is the cOAlition S initiative announced in September 2018 by a group of research funders, which mandated research supported by public grants to be published without embargo in open-access journals or platforms starting in 2021, as laid out in Plan S (71). Similarly, the U.S. Office of Science and Technology (OSTP) issued a policy memo in August 2022 to federal departments and agencies, requiring federally funded research to be made freely available without delay (72). The rise of Plan S and other funder mandates stipulating that articles must be published under an open license has required publishers to pivot from a traditional subscription-based publication model to accommodate these new requirements. One major development in this area has been the rapid rise of transformative agreements between journals or publishers and authors’ institutions, in which payments from institutional subscribers are applied toward open-access publication costs instead of journal access paywalls (73). However, Plan S support for these transformative arrangements will cease as of 31 December 31 2024, and Plan S as well as other funder mandates do not financially support publication in so-called hybrid journals that publish both subscription and open access content. Publishers are responding by seeking alternative open access publication routes. One such example is the subscribe-to-open (S2O) model that requires yearly subscription thresholds to be met for journal content from that year to be published under an open access license. The journal *Annual Reviews* pioneered this model, and its advantages include reliance on a publisher’s existing subscriber base and compliance with funder OA policies (74). In July 2023, ASM announced that it will be converting its six primary research subscription-based journals to a subscribe-to-open model in 2025 (75). As these developments illustrate, the open access movement over the past three decades has significantly disrupted the scientific publishing enterprise and required adaptation by publishers, authors, and other stakeholders that will continue for the foreseeable future.

### Diversification of journal types

The original purpose of journals was to communicate research findings to inform the scientific community and establish priority of discoveries; therefore, journals primarily published research articles. Diversification has occurred not only in terms of the types of articles published, such as original research, reviews, and perspectives, but also in terms of the types of journals available to researchers. Journals now specialize in the types and formats of information they report, as well as in scope and access. For example, ASM recently initiated *ASM Case Reports* as a new home for case reports in clinical microbiology and infectious diseases (76). This journal will become the primary venue for the reporting of individual illustrative patient cases, complementing *Antimicrobial Agents and Chemotherapy* and the *Journal of Clinical Microbiology*. Other specialized journals at ASM include *Microbiology Research Announcements,* which informs the community of microbiological resources newly placed into public repositories, and *Microbiology Spectrum,* a journal with a broad scope that includes not only novel research findings but also replication studies, validation studies, negative results, and methodological advances, provided that the work is technically sound. Examples of specialized journals outside of ASM include journals focused on the publication of short communications and those publishing novel methods.

### Educating communities

Another recent development among scientific journals and publishers is to offer content beyond journal articles, such as webinars and podcasts that highlight published research or authors, which are generally free of charge. At ASM, the journal *mSystems* hosts “Thinking Series Webinars” throughout the year, in which editors discuss topics related to systems microbiology with invited speakers and engage with attendees via a Q&A section (77). The *Journal of Microbiology and Biology Education* (JMBE) hosts live webinars (78) that feature discussions with authors of recently published articles. ASM Journals has an official podcast called “Editors in Conversation” (79), in which ASM journal editors, researchers, and clinicians address recent issues in the microbial sciences, as well as a variety of podcast series aimed at making microbiology more accessible to a broader audience, including a Spanish language series called “El Mundo de los Microbios” (80). To further increase accessibility, many ASM journal articles feature an “importance” section that summarizes the paper’s content and significance in lay terms. Other publishers and journals provide similar services to their communities. For example, the *Nature* journals portfolio has weekly podcasts in which newly published research is highlighted and discussed (81), and Cell Press provides webinars moderated by editors in which recent scientific developments are showcased (82). Some articles published in *The Lancet Microbe* feature an accompanying audio of conversations between editors and authors (83). Similarly, the ASM journal *mSystems* invites authors of accepted original research articles to submit a 1-minute video about their paper, which allows junior authors to receive greater exposure for their work (84).

Showcasing journal article content via these alternative routes benefits the scientific community in many ways. First, it increases the visibility of research findings in journal articles and the scientists who performed the work. Second, webinars and podcasts provide accessible presentations of content, which may otherwise be too complex for non-scientists or scientists unfamiliar with a particular field to understand. By listening to editors and authors discuss the research presented in journal articles, listeners can learn how authors approached a problem, what they found, and how they dealt with challenges, thus enhancing their understanding of the scientific process.

### The rise of artificial intelligence (AI)

The rise of generative AI tools since late 2022 has created new challenges for scholarly publishing. These tools can create content, including text and images, that can be difficult to distinguish from content authored by humans, and their use stands in conflict with many journal policies stating that all content in submitted manuscripts must be produced by the authors. Some of the challenges that generative AI presents to the research community include determining the authenticity of content, reporting the use of AI, and recognizing and addressing biases underlying AI algorithms and training data (85). Another issue is the use of generative AI tools for peer review since any documents that reviewers receive are strictly confidential, and confidentiality would be compromised if reviewers used AI tools to craft their feedback. Publishers and journals have responded to the rapid expansion of AI tools by creating policies regarding the use of generative AI. At ASM journals, the use of AI tools must be disclosed, and content entirely created by AI tools without significant human oversight is prohibited (86). The release and rapid adoption of AI tools has added to journals’ responsibilities to ensure integrity of their published output by putting new policies in place, screening for AI-generated content, and responding to ethical concerns regarding the use of AI tools in published articles.

### Conclusions

After more than three centuries of relative stability in which scholarly journals served as gatekeepers of scientific communication, journals have begun to assume a proactive role to maintain the integrity of the research literature, through actions ranging from peer review education to serving as repositories for primary data. Much of the time required to submit and publish a paper reflects the complex responsibilities of modern journals for enforcing the norms of science, verifying authorship, ensuring compliance with ethical guidelines, verifying data formatting and availability, and above all, certifying the validity of the research. Although scholarly publishing is presently under stress from changing economic models, the misuse of impact factors, the rise of predatory journals, and an epidemic of retractions, one cannot help but marvel at the remarkable ongoing historical changes regarding the role of journals that will have profound consequences for the future of science. Journals are at the vanguard of a movement in which science is reforming itself to enhance communication and improve the quality of the scientific literature. Scientists must join in supporting the ongoing evolution of scientific publishing so that journals can continue to “curate, catalyze, and clarify” research advances (1).
